# Surveillance for Action: Operationalizing Private Sector Surveillance and Service Delivery across the Malaria Transmission Continuum

**DOI:** 10.4269/ajtmh.23-0447

**Published:** 2024-04-23

**Authors:** Erica Berlin, Christopher Lourenço, Stephen Poyer

**Affiliations:** PSI, Washington, District of Columbia

## Abstract

Despite global recommendations that surveillance systems should capture malaria case data from both private and public sectors, the integration of private sector data into national systems remains a challenge for national malaria programs in high-burden settings. The WHO’s *Malaria Surveillance, Monitoring & Evaluation: A Reference Manual* suggests eight general guidelines for conducting private sector surveillance. Practical operational guidance is needed to implement private sector surveillance and service delivery interventions. This commentary describes available evidence and lessons learned from the Greater Mekong subregion and sub-Saharan Africa on mapping and geolocating private providers; adding private providers to national digital registries; strengthening regulation, training, and supervision; providing feedback; and subsidizing commodities. Reporting tools should be electronic, aligned or integrated with existing national systems, and adapted to meet private sector needs. A strong enabling environment is also required, including regulatory systems that mandate private providers be registered and report into national surveillance systems. Where possible, existing systems and government personnel should be used to train, supervise, and mentor private providers. Commodities such as quality-assured artemisinin combination therapies and rapid diagnostic tests need to be available to clients at affordable prices. Finally, any parallel private sector surveillance systems need to be incorporated into the Ministry of Health systems, and the Ministry of Health needs to regularly engage with the private sector. However, the private sector contains a broad spectrum of provider types and varies across countries. Strategies need to be adapted to local contexts.

## INTRODUCTION

In many high-burden malaria countries in sub-Saharan Africa, a substantial percentage of individuals first seek care for illness from private-sector providers. National representative surveys from 2015 to 2017 found that more than half of caregivers of febrile children in Chad, Democratic Republic of the Congo, Ghana, Nigeria, and Uganda sought initial treatment in the private sector.^1^ However, the private sector has historically been left out of government oversight and supervision activities, with limited national resources being focused on the public sector.^2^ As a result, private sector reporting into national health information systems is often limited or inconsistent, and routine data on service delivery practices and quality are lacking.^2^ To identify the true transmission burden and support appropriate responses, surveillance systems must be able to detect malaria cases across the diverse cadres of healthcare providers, not just from public or formal sectors.^2^

The WHO’s *Malaria Surveillance, Monitoring and Evaluation: A Reference Manual* suggests eight general guidelines to support countries in improving private sector malaria surveillance and, with it, service delivery quality.^2^ However, the reference manual lacks practical operational guidance on how to engage the wide variety of actors involved in private sector healthcare in support of the guidelines. This commentary describes available evidence and lessons learned in several sub-Saharan African and Greater Mekong subregion settings for each of the eight private sector guidelines. We examine best practices in operationalizing the guidelines and explore challenges that can affect the feasibility of adopting these strategies. We conclude with recommendations for improving private sector surveillance across the eight domains.

### Mapping private healthcare providers.

In countries where private sector reporting has not started or has been limited, the first step should be generating a list of providers whose services include malaria management, covering outlet type, location, regulation status, level of reporting, internet connectivity, and other factors relevant in this setting. Collaboration with regulators (e.g., food and drug regulators), professional organizations, or community-based organizations that already maintain lists of registered providers can facilitate this process. Once an initial private provider list has been established, it can be expanded through conducting a targeted census of outlets to capture more details on known providers and add records for currently unlisted providers. The Greater Mekong Elimination of Malaria through Surveillance (GEMS) program in Cambodia, Lao People’s Democratic Republic, Myanmar, and Vietnam began by conducting a comprehensive census of formal and informal private sector providers that were missing from government lists. Providers were mapped and enrolled into a private sector malaria case management and surveillance program. The lack of complete, up-to-date government registries of formal private providers and very limited information on informal providers meant a labor- and resource-intensive process. In this setting, the number of open outlets fluctuated between seasons, with temporary outlet closures or relocations complicating the task of maintaining accurate lists over time. A lesson from this mapping exercise is to start early in collaboration with key government stakeholders and recognize that dedicated time and resources may impede other activities.^3^

### Developing databases of private healthcare providers.

Once a list of private providers who treat malaria has been established or updated, the private provider list should be added to the country’s Common Geo-Registry if one exists or to the hierarchy of the Health Management Information System (HMIS). If it is not possible to add private providers into the national registry or facility list, the private provider list could be maintained through a parallel database in DHIS2, MS Excel, or other platform; however, this is more likely to cause fragmentation, reduce operational efficiencies, and create additional barriers to system sustainability. Ideally, each provider record should be geocoded. If an outlet census is conducted to map private providers, it is often possible to geocode sites then.

### Exploring approaches to strengthening regulation for registration and reporting.

A review by Bennett et al.^4^ found that regulation through licensing and accreditation of private sector providers in Tanzania to create accredited drug dispensing outlets (ADDOs) helped improve artemisinin-based combination therapy (ACT) supply by enforcing diagnosis and treatment protocols, creating price controls, and assuring drug quality standards. However, the review found evidence from multiple countries that in relation to the private sector, infrequent inspections, ineffective sanctions, and low enforcement are frequent regulation gaps. Provider regulation lies above the remit of any one health area within the Ministry of Health. Cross-department leadership will help leverage experiences and funding from multiple disease programs toward the objective of a more effective private healthcare sector for all. In elimination settings, malaria is typically a notifiable disease, and the main challenges center around enforcing the legal requirement of mandatory reporting. Approaches for formalizing and regulating private providers must include road maps with clear roles and responsibilities and timelines for how regulations will be enforced. Engagement on regulation could take place in parallel with surveillance efforts. For example, registration fees could initially be waived in return for active participation in case reporting.

### Studying approaches and incentives for improving provider performance.

Appropriate provider behaviors underpin quality fever case management irrespective of sector, but drivers of behavior may differ by cadre and country. Programs can use behavioral insights to understand and improve provider service delivery behaviors. Breakthrough ACTION has developed a framework for provider behavior change for malaria. A framework such as this can be used to identify and target priority provider behaviors such as adherence to test results, particularly minimizing the treatment of patients who test negative.^5^

Evidence from the GEMS project showed that drivers of private provider motivation can vary across geographies and by provider characteristics. For example, providers who said they would not maintain malaria test and treatment stocks after the program ended had lower levels of internal motivation in Myanmar and lower levels of internal and external motivation in Vietnam. Overall, providers were motivated by a variety of personal factors and, notedly, were not solely financially motivated. Countries should conduct similar provider motivation assessments in collaboration with professional associations to understand relevant drivers of behavior in their setting.^6^ Frameworks for behavior change can then be used to design, implement, and evaluate provider behavior change interventions.

### Ensuring routine interaction between Ministry of Health and private healthcare providers.

Incorporating private sector providers into routine government activities such as malaria training, supportive supervision, and information dissemination provides a sustainable platform to strengthen private provider service delivery and data reporting.^7^ Evidence from ADDOs in Tanzania showed that training and supervision can improve malaria testing and provider adherence to test results.^8^

#### Training.

The review by Bennett et al.^4^ identified training of private sector providers as one of the more operationally feasible and cost-effective ways to improve private provider adherence to antimalarial prescription guidelines. However, there was evidence of increased effectiveness in some settings when training was combined with one or more complementary interventions such as referral systems, regulatory oversight, and behavior change communication. Stakeholder engagement meetings at the end of a Unitaid-funded project to create a private sector market for rapid diagnostic tests (RDTs) in Nigeria found that even if private providers were trained and had standard operating procedures for RDT administration, many still prescribed ACTs to people who were not tested or had negative test results. Stakeholders thought this could be addressed through more retraining and closer supervision.^7^

However, classroom-based training may be challenging for private providers to attend. They may lose income if they need to leave their site during operating hours, and multiple in-person sessions may not fit with providers’ business schedules. Experience in countries such as Angola have shown that innovative solutions such as training during nonpeak hours or self-paced digitalized trainings may be effective. In Angola, the USAID-funded Health for All project has developed and used an e-learning platform called Kassai to train public and private providers on malaria and other health areas, with access provided through any internet-enabled device.^9^^,^^10^ This type of training could provide cross-cutting benefits through integrated health systems strengthening on several health areas at once and reduce training silos for both public and private providers. To ensure that knowledge is retained and providers continue to adhere to appropriate behaviors, training must be accompanied by follow-up interventions and monitoring.

#### Supervision.

Countries already benefiting from digitalized supervision checklists for public health facilities, such as those implemented through Outreach Training and Supportive Supervision Plus with the support of the U.S. President’s Malaria Initiative’s Impact Malaria project, can adapt existing digital tools for private providers. Experiences from more than 29 countries that have used this supervision approach have shown that supervision should address the work environment, RDT procedures, treatment procedures, referral procedures, case management competencies, and data reporting. Supervision techniques should include direct observation of procedures, verification of recorded data, identification of challenges, provision of on-the-job training and mentorship, provision and documentation of recommendations for improvement, and follow-up to ensure that corrective measures are implemented. If it is not possible to train and supervise all private providers, available data should be used to prioritize providers for support. Providers could be ranked based on their malaria reporting rate and client load, or if they are not yet reporting data, based on population census data and subnational epidemiological data from the public HMIS.

Government stakeholders in Nigeria felt that it is critical to build the capacity of designated focal points for monitoring and evaluation and supportive supervision at the local government and district levels. Building their capacity will enable them to extend effective supervision to the private sector and integrate this work with their established data monitoring and supervision systems.^7^ Countries should identify opportunities to integrate private sector malaria supervision activities with supervision for other disease areas as part of increasing efforts to build resilient and sustainable systems for health.

### Providing simple, inexpensive reporting systems.

The WHO recommends that private sector providers should be provided with simple, inexpensive reporting tools and systems.^2^ The private sector reporting system should be the same as that for public providers, or integrated to the furthest extent possible. However, the most feasible reporting approaches for private providers may vary, and countries should select the approach that is most appropriate across their channels and contexts. The ideal reporting approach may also vary over time; for example, a reduced set of data elements could be used initially when rolling out a new system. If it is not possible to find a solution that works for all outlets, countries should select a solution that works for the most frequented or epidemiologically important private providers and locations. Lessons learned from the GEMS project and the Unitaid-funded RDT project in Nigeria were that paper-based data collection can be slow and tedious and may interfere with timely care provision. On the other hand, digital reporting systems enable rapid availability of data throughout the health system and can provide a better user experience; however, they also introduce challenges such as connectivity, software errors, and the need to maintain and update software over time.^11^ In GEMS countries, case reporting, data collection tools, and data flows for the private sector were co-designed by monitoring and evaluation experts and National Malaria Control Program (NMCP) staff to align with countries’ standard forms and processes and enable integration with government systems. Then, midway through implementation, mobile case reporting was introduced in three of the countries, using DHIS2 to capture, store, and validate data before they were submitted to the NMCPs.^3^ When considering private sector integration with government systems, a key challenge can be building enough confidence in the quality of the data such that public sector leaders are willing to integrate. In GEMS, routine data quality assessments helped build NMCP confidence over time and eventually showed that accuracy of malaria case data reported from the private sector was >95% and that data were generally complete.^11^ Continuous data monitoring and frequent data quality assessments should be conducted to help increase confidence in private sector data over time and improve data use.

### Ensuring consistent feedback to providers.

Once ties have been strengthened between the Ministry of Health and private providers, training, supervision, and mentorship visits all provide opportunities for provider feedback on performance. Prior to the establishment of routine private sector supervision systems, feedback, comments, or other concerns can be raised with professional organizations acting as representatives of their private sector members. Once digital reporting systems are in place, countries can explore sending ad hoc or routine feedback to providers through push notifications or text messages. If DHIS2 or a similar platform is used, it is possible to create dashboards that private providers can view to track their performance.

### Obtaining subsidized or free commodities.

Wide-scale ACT subsidies began in 2010 through the Affordable Medicines Facility-malaria (AMFm) program established by the Global Fund to Fight AIDS, Tuberculosis and Malaria. By 2012, AMFm subsidies led to substantial price reductions of quality assured artemisinin-based combination therapies (QAACTs) in six of the eight pilot settings and an increase in the availability of ACTs. The AMFm was followed in 2013 by the Global Fund’s private sector co-payment mechanism (CPM). An ACTwatch study in 2017 showed that during the CPM program, there continued to be significant increases in QAACT availability in the private sector in four of five countries.^12^ More recent descriptions of national market changes are hampered by the lack of contemporary private sector market data. Introducing RDT subsidies could potentially lead to similar changes.^4^ It is likely that in many settings, introducing or maintaining private sector subsidies for key malaria commodities could create an enabling environment for continued gains in ACT availability and improvements in RDT availability.

## CONCLUSION

Diverse experiences across malaria burden settings have shown that effective private sector surveillance requires mapping and geolocating private sector providers, adding private providers to national digital registries, and creating simple reporting mechanisms. Lessons learned have shown that reporting tools should be digitalized, aligned, or integrated with existing systems in a country and adapted to the needs of private providers. Regulatory pathways must mandate private provider registration and support an enabling environment for incorporating private sector reporting into national surveillance systems. In addition, commodities such as QAACTs and RDTs need to be available to providers and their clients at affordable prices through subsidies or other incentives. Lastly, the private sector surveillance system needs to be incorporated into Ministry of Health systems, and the Ministry of Health needs to regularly engage with private sector providers and their professional representatives. Where possible, existing systems and personnel should be used to train, supervise, and mentor private providers and maintain reporting systems. The eight WHO guidelines for improving private sector surveillance and quality service delivery may be simple, but implementing them is no easy task and will require continued engagement and resources and sharing of experiences as countries continue the challenging road to malaria elimination.

## References

[1] World Health Organization, 2019. Meeting Report of the WHO Technical Consultation on Malaria Case Management in the Private Sector in High-Burden Countries. Geneva, Switzerland: World Health Organization. Available at: https://www.who.int/publications/m/item/WHO-CDS-GMP-MPAC-2019.12. Accessed May 19, 2023.

[2] World Health Organization, 2018. Malaria Surveillance, Monitoring & Evaluation: A Reference Manual. Geneva, Switzerland: World Health Organization.

[3] Potter R, , 2023. Private sector contributions to national malaria surveillance systems in elimination settings: Lessons learned from Cambodia, Lao PDR, Myanmar, and Vietnam. Am J Trop Med Hyg 108 *(Suppl):* 14–23. 35914687 10.4269/ajtmh.22-0147 PMC9904159

[4] Bennett A Avancena ALV Wegreit J Cotter C Roberts K Gosling R, 2017. Engaging the private sector in malaria surveillance: A review of strategies and recommendations for elimination settings. Malar J 16: 252. 28615026 10.1186/s12936-017-1901-1 PMC5471855

[5] Breakthrough Action, PMI Impact Malaria, 2020. *A Blueprint for Applying Behavioral Insights to Malaria Service Delivery: Methods and Frameworks for Improving Provider Behavior*. Available at: https://breakthroughactionandresearch.org/wp-content/uploads/2020/06/Blueprint-for-Applying-Behavioral-Insights-to-Malaria-Service-Delivery.pdf. Accessed May 18, 2023.

[6] Brown M Bouanchaud P Tesfazghi K Phanalasy S Thet MM Nguyen H Wheeler J, 2022. Motivation to test, treat, and report malaria cases: A quantitative assessment among private sector providers in the greater Mekong subregion. Malar J 21: 82. 35264168 10.1186/s12936-022-04108-7 PMC8905864

[7] Odugbemi B, , 2018. Private sector malaria RDT initiative in Nigeria: Lessons from an end-of-project stakeholder engagement meeting. Malar J 17: 70. 29409502 10.1186/s12936-018-2222-8 PMC5801847

[8] Maloney K, , 2017. Expanding access to parasite-based malaria diagnosis through retail drug shops in Tanzania: Evidence from a randomized trial and implications for treatment. Malar J 16: 6. 28049481 10.1186/s12936-016-1658-y PMC5209819

[9] U.S. President’s Malaria Initiative, 2022. *Video: USAID Introduces Digital Technology for Healthcare Professionals in Angola*. Available at: https://www.pmi.gov/video-usaid-introduces-digital-technology-for-healthcare-professionals-in-angola/#. Accessed June 14, 2023.

[10] PSI, 2021. *Efficient and Convenient Remote Capacity Building of Frontline Health Workers in Angola*. Available at: https://www.psi.org/2021/03/efficient-and-convenient-remote-capacity-building-of-frontline-health-workers-in-angola/. Accessed June 14, 2023.

[11] PSI, 2023. *GEMS+ Health System Strengthening Through Private Sector Engagement Lesson Learned from Malaria Elimination: The Learning Legacy of the Greater Mekong Subregion Elimination of Malaria Through Surveillance Project 2016–2022*. Available at: https://www.psi.org/wp-content/uploads/2023/09/GEMS-Legacy-Book-2023.pdf. Accessed January 22, 2023.

[12] ACTwatch Group; Tougher S Hanson K Goodman C, 2017. What happened to anti-malarial markets after the Affordable Medicines Facility-Malaria pilot? Trends in ACT availability, price and market share from five African countries under continuation of the private sector co-payment mechanism. Malar J 16: 173. 28441956 10.1186/s12936-017-1814-z PMC5405529

